# 3D-Printed Titanium Cages for Anterior and Lateral Lumbar Interbody Fusion Result in Excellent Fusion Rates One Year After Surgery

**DOI:** 10.1177/21925682251344557

**Published:** 2025-06-01

**Authors:** Anna-Katharina Calek, Bettina Hochreiter, Aaron J. Buckland

**Affiliations:** 1 72554 Melbourne Orthopaedic Group, Melbourne, VIC, Australia; 2 Department of Orthopedics, Balgrist University Hospital, University of Zurich, Zurich, Switzerland; 3 Spine and Scoliosis Research Associates Australia, Melbourne, VIC, Australia

**Keywords:** 3D-printed, titanium, cage, interbody fusion, ALIF, LLIF, fusion

## Abstract

**Study Design:**

Retrospective study.

**Objective:**

To determine the fusion rate in patients undergoing anterior lumbar interbody fusion (ALIF) and/or lateral lumbar interbody fusion (LLIF) with titanium cages.

**Methods:**

Fusion at 1-year was assessed by computed tomography (CT) using Lenke-Bridwell classification. Flexion-extension lateral radiographs confirmed fusion if motion was <5° through the fused segment. Perioperative metrics including bone graft type, operative time, estimated blood loss, revisions within the first postoperative year, clinical outcome assessed by the Oswestry Disability Index (ODI).

**Results:**

One hundred patients with a total of 137 fusion levels with 3DPTi cages were identified. In this cohort, 75% underwent primary surgery and 25% had a previous surgery. At 1-year post-op, 97.1% of interbody levels were fused (Grade I) on CT, and all levels appeared fused on flexion-extension radiographs. Four patients (4%) required additional surgery within the first two years. No revisions were required for cage subsidence/migration, or pseudoarthrosis. Median ODI significantly improved from 39 at baseline to 10 at 1-year (*P* = .001).

**Conclusion:**

3D-printed titanium cages for ALIF and LLIF result in excellent fusion rates at one year postoperatively without the use of rhBMP-2.

## Introduction

The use of anterior lumbar interbody fusion (ALIF) and lateral lumbar interbody fusion (LLIF) has increased in recent years due to their minimally invasive technique and efficacy in restoring coronal and sagittal alignment.^1-3^ Complications associated with interbody cages, such as subsidence, migration, and pseudarthrosis can result in loss of alignment, neurological compression, early screw loosening and recurrent pain 4. In addition to patient factors, such as low bone density, older age, female gender, and technical factors, such as endplate violation, that can negatively affect the outcome,^5,6^ cage materials play an important role.

Since the late 1990s, polyetheretherketone (PEEK) cages have been commonly used because of ease of ability to assess fusion, in addition to a modulus of elasticity closer to that of cortical bone than other biomaterials.^7^ However, PEEK does not allow material integration with bone and may be associated with cage migration and pseudoarthrosis.^8^ Engineered to reduce complications related to the interbody device itself, three-dimensional printed titanium (3DPTi) cages have recently been introduced.^9^ 3DPTi cages were developed after prior successful orthopaedic applications, where improvements in geometry that maximizes bone-to-implant contact, a surface roughness and porosity that provides better initial interference fit, and promote bone on-growth and in-growth. Whereas titanium is likely to be more osteoconductive and bioactive,^10^ and its superiority over PEEK cages in terms of fusion rate has been demonstrated in posterior-based spinal fusion,^11-13^ the reports of titanium cages in anterior- or lateral-based spinal fusion are scarce.

The purpose of this study was to analyze the fusion rates of ALIF and LLIF utilizing 3DPTi cages assessed with computed tomography (CT) scans and flexion-extension lateral radiographs 1-year postoperatively in patients with adult degenerative pathologies.

## Materials and Methods

Patient data was reviewed from an institutional review board approved, prospectively and consecutively enrolled, single surgeon registry of adult patients in Australia between 2020 and 2024. Ethics committee approval was obtained before commencement of this study, and informed consent was obtained from each patient. Inclusion criteria included the use of 3DPTi cages (Modulus ALIF & Modulus XLIF; NuVasive Inc, San Diego, CA, USA) in skeletally mature patients undergoing lumbar spinal fusion for degenerative pathologies between L1 and S1. CT scan was performed at 1-year follow-up per standard of care. Patients treated with different cage designs, and for infection, fracture or tumor were excluded. The choice of approach was based on the patient’s anatomy as assessed by preoperative scans and previous surgery: while ALIF was used for all L5-S1 levels, LLIF or ALIF was used for the L4-5 level.^14^ All surgeries were performed by the senior author.

The primary endpoint of the study was the rate of fusion at 1-year assessed on CT scans by a fellowship-trained orthopaedic surgeon. Fusion was graded according to Lenke-Bridwell et al through the interbody space.^15^ Furthermore, flexion-extension lateral radiographs were assessed at 1-year postoperatively and fusion confirmed if < 5° range of motion was detected through the fused segment.^16^ Furthermore, bone graft use, surgical time, estimated blood loss, intraoperative complications, revisions within the first two postoperative years, and clinical outcome as assessed by the Oswestry Disability Index (ODI) at 1-year postoperatively were analyzed.

## Statistical Analysis

Statistical analysis was performed in a descriptive fashion. Numeric variables were expressed as mean (±SD) or median (interquartile range (IQR)) according to data distribution and discrete outcomes as absolute and relative (%) frequencies. Normality was assessed with the Shapiro-Wilk test. Repeated-measures analyses were performed with Friedman’s test. If the null hypothesis of Friedman’s test was rejected, post-hoc pairwise analyses were performed with Nemenyi’s test. Alpha risk was set to 5% (α = 0.05). Statistical analysis was performed with EasyMedStat (version 3.30; https://www.easymedstat.com/).

## Results

### Patient Characteristics

One hundred patients with a total of 137 levels were analyzed. In all considered levels a 3DPTi cage was used. Thirty-four (34%) patients were male and the median age was 65.8 years at surgery. One patient was a former smoker and there were no current smokers included in the cohort.

Further information on demographics and surgical characteristics is shown in Table 1.

**Table 1.** Demographics and Surgical Characteristics.

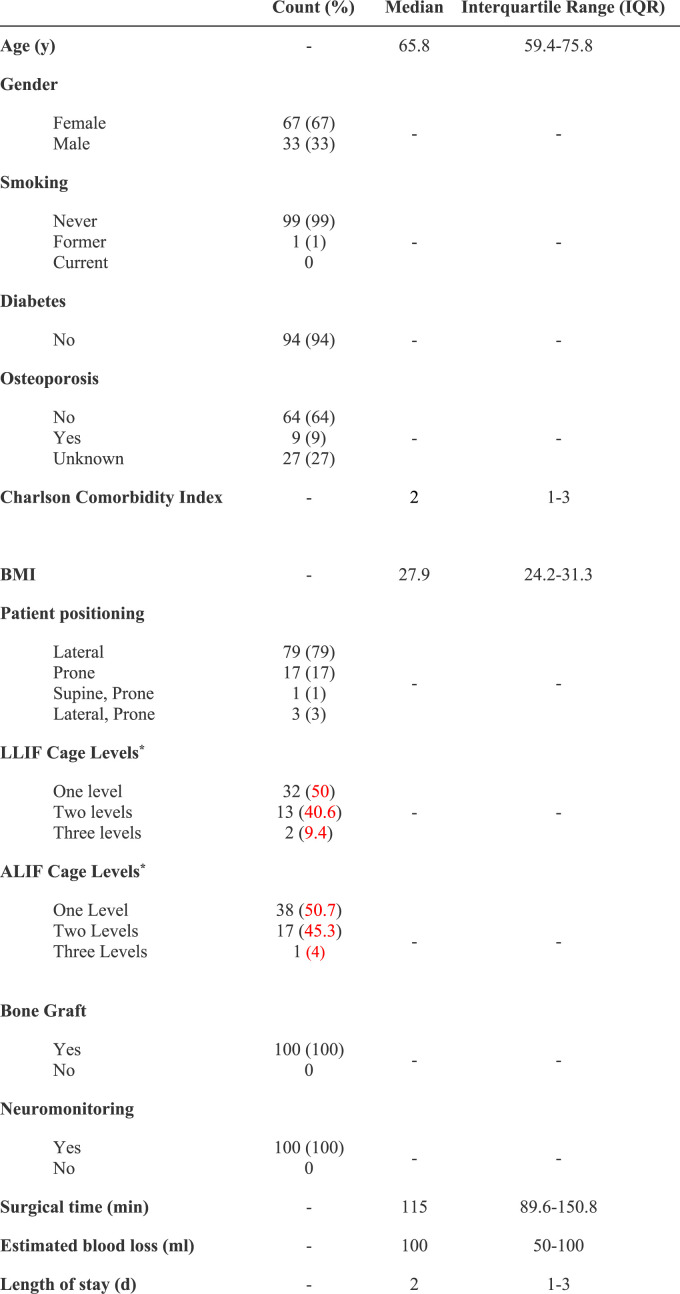

### Surgical Characteristics

Fifty-one (51%) patients underwent spinal fusion for degenerative spondylolisthesis, 13 (13%) patients for adjacent segment disease, 19 (19%) patients for discogenic back pain, 15 (15%) patients for foraminal stenosis, two patients (2%) for facet arthropathy. Twenty-five patients underwent a revision procedure (decompression surgery or fusion surgery) and 75 patients a primary procedure. All patients had supplemental posterior instrumentation (89% percutaneous, 11% open), and 16% had direct decompression (81.3% tubular, 18.7% open).

The median operative time was 115 minutes (IQR: 89.6-150.8) with a median estimated blood loss of 100 mL (IQR: 50-100). The median length of hospital stay was 2 days (IQR: 1-3; Table 1).

### Bone Grafts

Bone grafts were used in all cages in all patients. A detailed list of the type of bone graft used in each case is shown in Table 2. In the majority of cases (97.9%), only demineralized bone matrix fiber (DBM fiber) or Bioglass Fiber was used for cage/disc space grafting. Eleven cases (11%) were performed open with grafting of the posterior fusion site, and 90 cases (89%) had bone graft placed only through the cage into the disc space.

**Table 2.** Bone Graft Characteristics.

Bone Graft	Count (%)
Demineralized bone matrix fiber (DBM fiber)	41 (41)
Bioglass fiber	47 (47)
DBM fiber + autograft local	9 (9)
Bone graft substitute (Tricalcium phosphate)	2 (2)
DBM fiber + local autograft + Bioglass Fiber	1 (1)

### Fusion Grading

**Figure 1.**
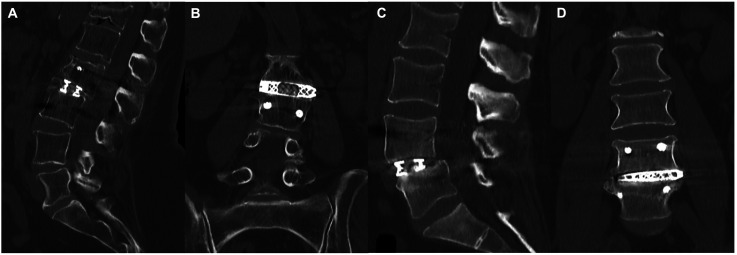
CT scans at 1-year postoperatively. A-B: A patient who underwent single-level LLIF L2/3 with a Grade I fusion at 1-year postoperatively. C-D: A patient who underwent a single-level LLIF L4/5 with a Grade II fusion (partial fusion) at 1-year postoperatively.

Overall interbody fusion rate (Grade I) was 97.1% of levels (133/137) at 1-year postoperatively on CT grading (Table 3). Two ALIF levels and two LLIF levels at L4/5 demonstrated intact grafts with incomplete remodeling (Grade II according to Lenke-Bridwell et al.^15^) at 1-year follow-up. The remaining ALIF and LLIF levels were Grade I (Figure 1A–B, Table 4).

**Table 3.** Total Fusion Grading.

Fusion Grading (ALIF + LLIF)^§^	Levels (%)
Grade I	135 (97.1)
Grade II	4 (2.9)

§according to Lenke-Bridwell et al (Grade I: complete fusion – fusion with remodeling; Grade II: partial fusion – intact graft with incomplete remodeling, no lucency present; Grade III: unipolar pseudoarthrosis, Grade IV: bipolar pseudoarthrosis).

Grades I and II were considered to be fused.

**Table 4.** Fusion Grading per Level After One Year.

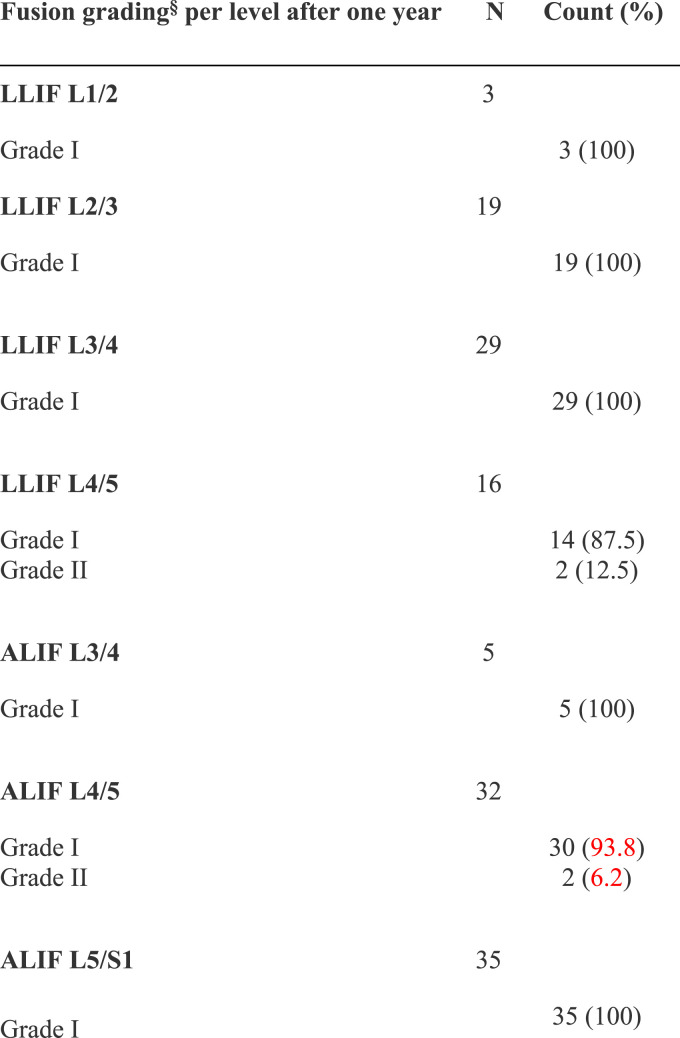

§according to Lenke-Bridwell et al (Grade I: complete fusion, Grade II: partial fusion, Grade III: unipolar pseudoarthrosis, Grade IV: bipolar pseudoarthrosis).

92.7% of fusion levels (127/137) were assessed with lateral radiographs. On flexion-extension radiographs, all assessed levels were considered fused with a range of motion of less than two degrees (Table 5).

**Table 5.** Evaluation of Fusion on Lateral Radiographs: Segmental Cobb Angle was Assessed on Flexion-Extension Radiographs/Sitting-Standing EOS Scans and the Difference was Calculated.

Level	N	Mean (SD)	95% CI	Range
L1/2	3	0.16° (0.47°)	0.14°-0.43°	0.38°-0.77°
L2/3	16	0.28° (0.9°)	−0.09°-0.77°	−1.6°-1.7°
L3/4	27	0.28° (0.98°)	−0.42°-1.06°	−1.74°-1.9°
L4/5	51	0.41° (0.85°)	0.19°-1.03°	−2.06°-1.9°
L5/S1	32	0.56° (0.94°)	−0.04°-1.25°	−1.65°-1.85°

### Complications and Revisions

There were three intraoperative complications: two vascular injuries (common iliac vein) repaired with sutures, and one durotomy repaired with sutures and fibrin glue. Four patients (4%) required an unplanned reoperation within the first two years after the index surgery. However, no revision was required due to cage subsidence, migration, or pseudoarthrosis. The reasons and timings for reoperation are included in Table 6.

**Table 6.** Reasons and Timings for Reoperation.

Reasons for Reoperation	
Pedicle screw malposition	2
Adjacent segment disease	1
Persistent foraminal stenosis	1
Timing for reoperation*	
within the first 90 days	2
between 90 days and two years	2

^*^after the index procedure.

### Patient Reported Outcomes

**Figure 2.**
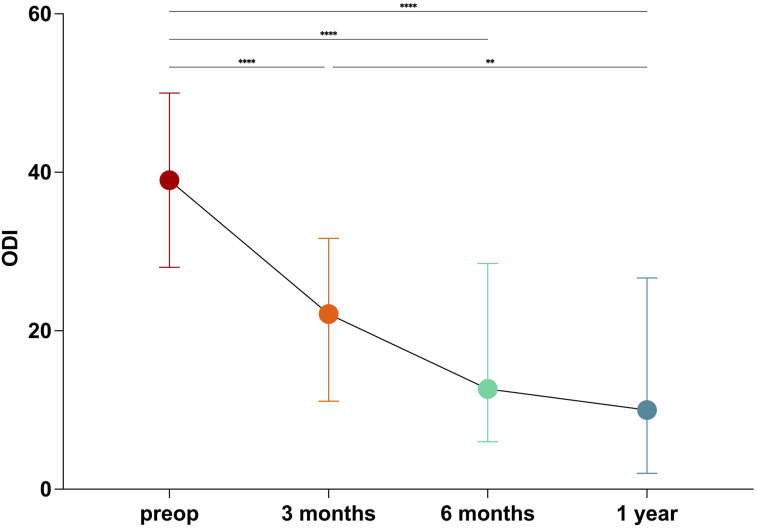
ODI over time after lumbar fusion surgery. At 6-month follow-up, the ODI plateaued without further statistically significant improvement. The whiskers show the minimum and maximum values, the median is shown as a dot. The line connecting the median values graphically depicts the decrease in the median ODI over time. ***P < 0.001; **P = 0.01.

The median preoperative ODI was 39 points (IQR: 28.2-49.5). The median ODI significantly improved at 1-year postoperatively compared to baseline: 1-year ODI: 10 (IQR: 2-26.5), *P* = .001. There was no significant change in ODI after 6 months: 6 months vs 1 year postoperatively: 12.7 vs 10.0, *P* = .759 (Figure 2). The ODI showed no significant change in the further course (1 year vs 2 years).

## Discussion

This study demonstrates that using 3D-printed titanium cages for ALIF and LLIF in patients with single-, two-, or three-level fusions results in a 97.1% (Grade I and II) fusion rate as observed on CT imaging 1-year postoperatively.

This high fusion rate is likely attributed to the cage material and a combined anterior/lateral and posterior approach. Titanium is an excellent material for interbody devices due to its strength and biocompatibility.^17^ This finding aligns with Malone et al.,^18^ who reported a similar fusion rate of 99.3% at 1-year postoperatively in patients undergoing LLIF with 3DPTi cages, underscoring the advantages of these interbody devices using tricalcium phosphate (TCP) as a bone graft substitute. Criticism of the use of TCP as a graft are that the radiopacity seen on imaging at 1-year may be a reflection of TCP that has not resorbed, and not of bridging bone.^19^ Our fusion rates are supportive of the findings of Malone et al^18^ albeit using DBM fibres or bioglass putty rather than TCP.

With improvements in geometry and porous surface, the biomechanical mismatch between implant and bone has been addressed,^20^ creating ideal conditions for solid fusion. Additionally, the use of ALIF and/or LLIF as fusion techniques has advantages over open posterior based transforaminal lumbar interbody fusion (TLIF) or posterior lumbar interbody fusion (PLIF). These techniques use a large interbody cage with a wide footprint aperture for graft material, which indirectly decompresses neural structures through ligamentotaxis and realignment. This approach not only reduces procedural morbidity with less complications such as cerebrospinal fluid leakage, but also increases fusion rates. Comparable titanium PLIF or TLIF cages result in fusion rates ranging from 83.3% to 93%.^11-13,21,22^

Despite this, one must not overlook that anterior and lateral procedures can be accompanied by complications that do not occur in posterior-based fusion surgeries, though modern techniques have reduced these risks significantly. LLIF, particularly at L4-L5, carries a risk of neurological complications such as femoral neuropraxia, thigh pain, and hip flexor weakness, but these are generally low (<2%) and rarely persistent when standardized approaches are used.^23^ ALIF procedures carry risks of vascular injury, retrograde ejaculation in males, bowel injury, and postoperative ileus.^24^ Single-position techniques for both approaches minimize complications by eliminating repositioning, reducing operative time and hospital stay while maintaining safety and radiological outcomes.^25^ Despite these advances, careful patient selection and anatomical planning remain essential to mitigate approach-specific risks.

Previous studies of ALIF and LLIF with PEEK interbody implants have reported satisfactory to good fusion rates ranging from 85% to 96.6%,^26-29^ which is considerably lower than the fusion rate found in this study. It should also be noted that the PEEK implants in the above-mentioned studies were used in combination with different biologics to increase the fusion rate and were evaluated according to different fusion grading criteria.

Unlike most other studies,^29-31^ in the present study, CT scans, which are more definitive, were used in combination with lateral flexion-extension or sitting-standing EOS scans to assess interbody fusion. On the CT scans, four levels demonstrated a Grade II fusion through the disc space after one year. These levels were in patients with poor preoperative bone mineral density, which inherently puts these patients at greater risk for subsidence or pseudoarthrosis.^32^ To reduce the risk of such complications, preoperative teriparatide treatment should be considered, as it can improve volumetric bone mineral density and fine bone structure.^33^ However, none of these four patients required revision surgery due to symptomatic pseudoarthrosis or cage subsidence. The reoperation rate in the present cohort was 4%, mostly due to fusion-related complications such as adjacent segment degeneration, persistent stenosis after indirect decompression, or screw malposition.

Assessment of the results of the radiographic evaluation, all levels evaluated were considered fused, with less than two degrees of range of motion detected between flexion and extension or sitting and standing. This finding is consistent with previous studies that have also highlighted the limitations of radiographs in accurately assessing fusion. Radiographs often result in higher false positive rates for fusion compared to CT scans. Lee et al.^34^ and Santos et al.^35^ thus concluded that CT scans are the more reliable method for visualizing bridging bone. Nevertheless, we believe that the combined assessment of fusion by lateral radiographs and CT scan is valuable and may help to allay concerns that fusion rates are overstated by CT.

Iliac crest autograft is still widely considered the gold standard for lumbar fusion, as higher fusion rates have been reported than with the use of allograft or synthetic bone graft substitutes.^36^ However, harvesting iliac crest bone grafts is associated with donor-side morbidity and can result in persistent pain in up to 32% of cases,^37^ which led to a growing interest in alternative bone graft materials.^38^ Although the use of rhBMP-2 undoubtedly results in high fusion rates,^37,39^ the results of the present study suggest that its routine use is not necessary. Non-rhBMP grafts, particularly DBM or bioglass putty, combined with a titanium cage result in comparable fusion rates. This is relevant as rhBMP has been associated with adverse events such as heterotopic ossification, infection and wound seroma^40-42^ and is also associated with high costs. In the present study, rhBMP was not used due to the lack of availability in this country.

The median ODI significantly improved at 1-year postoperatively compared to baseline. Observing the range of the ODI, it covers a wide span at both time points. This observation is consistent with expectations given that a predominant number of patients underwent multilevel fusion procedures.

There are several potential limitations to this study. First, the study population was relatively heterogeneous with patients undergoing single-level, two- or three-level interbody fusion. While this diversity can be seen as a strength, as the results show excellent fusion rates across the study cohort, suggesting that 3D-printed titanium cages provide reliable fusion rates even for larger surgeries in frail patients with poor biology, it also introduces variability that may affect the generalizability of the results. Secondly, one could argue that the follow-up period was short, but the main aim of this study was to report on the fusion rate, which reached almost 100% at 1-year. In this context, an extended follow-up period would not yield additional insights regarding fusion rates. Third, this study reports the results of a single-surgeon practice, which does not necessarily imply external validity. However, it allows for a homogeneous approach in terms of indications and surgical technique, including the use of bone graft, which might otherwise introduce bias.

## Conclusion

3D-printed titanium cages for ALIF and LLIF result in excellent fusion rates at one year postoperatively without the use of rhBMP-2.

Disclaimer: The authors, their immediate families, and any research foundations with which they are affiliated have not received any financial payments or other benefits from any commercial entity related to the subject of this article.
